# Systemic and local eosinophil inflammation during the birch pollen season in allergic patients with predominant rhinitis or asthma

**DOI:** 10.1186/1476-7961-5-4

**Published:** 2007-10-29

**Authors:** Mary Kämpe, Gunnemar Stålenheim, Christer Janson, Ingrid Stolt, Marie Carlson

**Affiliations:** 1 Department of Medical Sciences, Respiratory Medicine and Allergology; University Hospital, Uppsala, Sweden; 2 Asthma Research Centre, University Hospital, Uppsala, Sweden; 3 Department of Medical Sciences, Gastroenterology Research Group, University Hospital, Uppsala, Sweden

## Abstract

**Background:**

The aim of the study was to investigate inflammation during the birch pollen season in patients with rhinitis or asthma.

**Methods:**

Subjects with birch pollen asthma (n = 7) or rhinitis (n = 9) and controls (n = 5) were studied before and during pollen seasons. Eosinophils (Eos), eosinophil cationic protein (ECP) and human neutrophil lipocalin were analysed.

**Results:**

Allergic asthmatics had a larger decline in FEV1 after inhaling hypertonic saline than patients with rhinitis (median) (-7.0 vs.-0.4%, p = 0.02). The asthmatics had a lower sesonal PEFR than the rhinitis group. The seasonal increase in B-Eos was higher among patients with asthma (+0.17 × 109/L) and rhinitis (+0.27 × 109/L) than among controls (+0.01 × 109/L, p = 0.01). Allergic asthmatics and patients with rhinitis had a larger increase in sputum ECP (+2180 and +310 μg/L) than the controls (-146 μg/L, p = 0.02). No significant differences in inflammatory parameters were found between the two groups of allergic patients.

**Conclusion:**

Patients with allergic asthma and rhinitis have the same degree of eosinophil inflammation. Despite this, only the asthmatic group experienced an impairment in lung function during the pollen season.

## Background

For many years, it has been known that allergic rhinitis and bronchial asthma co-exist [1]. The majority of patients with allergic asthma present with symptoms of seasonal or perennial rhinitis and, in epidemiological studies, rhinitis was found in up to 80%–100% of patients with asthma [2,3]. Perennial rhinitis has also been found to be a risk factor for asthma, independent of allergy [4] and bronchial responsiveness [5], and, moreover, airway remodelling is often present in non-asthmatic patients with allergic rhinitis [6,7].

This link between the upper and lower airways is also supported by experimental data from allergen challenges. Becky Kelly *et al*. have demonstrated a reduction in airway function and an airway inflammatory response after local antigen challenge in atopic patients with or without asthma [8]. Braunstahl and co-workers found that either allergen provocation of the nose or segmental bronchial provocation resulted in generalised airway inflammation [9,10]. Murine animal studies provide further evidence of the systemic link between nose and lungs [11,12]. Treatment studies also point in the same direction. Pedersen *et al*. [13] demonstrated an improvement in asthma in patients treated with nasal steroids for their rhinitis and a correlation between nasal eosinophils and a reduction in FEV_1 _in patients with perennial allergic rhinitis has been reported [14].

It is well established that the allergic inflammation in the nasal and bronchial mucosa is characterised by tissue eosinophilia. The allergic cascade and eosinophil migration is dependent on the expression of cytokines, chemokines and adhesion molecules. The eosinophils release their cytotoxic granule proteins as eosinophil cationic protein (ECP), myeloperoxidase, eosinophil protein X and eosinophil peroxides, causing airway damage and the remodelling of the airway [15,16]. The role of neutrophils in allergy and asthma has been discussed. According to GINA (updated 2004), non-allergic and allergic asthma are not distinct immunopathological entities. Recent studies have, however, shown that airway inflammation in chronic severe asthma displays an increased number of neutrophils [17]. Human neutrophil lipocain, HNL, which has been shown to be an entirely specific marker of neutrophils [18], can be used to detect neutrophil involvement.

There are still many questions to be resolved. Previous studies have mainly been performed with high-dose allergen challenge, which is not consistent with natural allergen exposure, although a few studies have used very-low-dose allergen challenge [19]. The aim of the present study was to investigate the inflammatory reaction during natural allergen exposure during the birch pollen season in birch-pollen-allergic patients with allergic rhinitis or allergic asthma as the predominant symptoms. Our hypothesis was that the location or the magnitude of the inflammatory response explains to some extent why some patients present with rhinitis and some with asthma, in spite of having the same levels of specific IgE antibodies to birch.

## Methods

### Patients

Sixteen birch-pollen-allergic patients were selected for the study. All these patients were skin prick test positive to birch pollen. None of the patients had symptoms during the winter and/or were on regular treatment for rhinitis or asthma outside the birch pollen season. Inhaled steroids or nasal steroids were not allowed out of season nor during the birch pollen season and none of the patients was on any other regular medication during season. None of the patients had smoked for the past ten years. Forced expiratory volume in one second (FEV_1_) out of season was more than 75% of predicted and FEV_1_/forced vital capacity (FVC) more than 70% in all patients. All the patients had doctors diagnosed seasonal allergic rhinitis or allergic asthma, diagnosed by a lung physician and allergologist at the allergy out-patient clinic. The patients with birch pollen induced asthma had a positive history of wheezing and dyspnea during pollen season, whereas patients whith allergic rhinitis all denied symptoms from the airways.

Seven patients that had a diagnosis of allergic asthma and reported having respiratory symptoms during the pollen season even when neither having a cold nor having exercised were categorised as having asthma as the predominant symptom (allergic asthma). Nine patients with a diagnosis of allergic rhinitis but not allergic asthma and having mainly eye and nose symptoms were categorised as having rhinitis as the predominant symptom (allergic rhinitis).

### Control group

The control group consisted of five healthy, non-atopic, never-smoking hospital employees and their relatives. These persons had no allergic symptoms either outside or during the birch pollen season. They were skin prick test negative to nine standard allergens, including birch pollen, had no IgE antibodies to birch and had normal lung function with an FEV_1 _of more than 80% of predicted.

### Study design

The study included three visits to our out-patient clinic and all the subjects were tested according to the procedure presented in Table 1. Both the inclusion visit and the baseline visit were performed out of season between November and February. When the pollen counts had reached 4,000 grains/m^3 ^of air, the patients were told to start recording their diary and, two to three weeks later, the season visit was made. The study was performed during the birch pollen seasons in 2000 and 2002, due to the low pollen count in 2001. Patients were included consecutively, thus all patients were studied pre-season and during season in the same year. The subjects were told to avoid short-acting bronchodilators and anti-histamines for 24 hours before the visit and nasal decongestants for four hours before the visit.

**Table 1:** Study design.

	Visit 1 Inclusion	Visit 2 Baseline out of season	Visit 3 Birch pollen season
Blood sample	x	x	x
Spirometry	x	x	x
Skin prick test	x		
Specific IgE for birch	x		
Nasal lavage		x	x
Induced sputum		x	x
Diary			
- *symptoms*			x
- *medication*			x
- *PEFR*			x

x: performed investigations at each visit

#### Total pollen count

**Figure 1:**
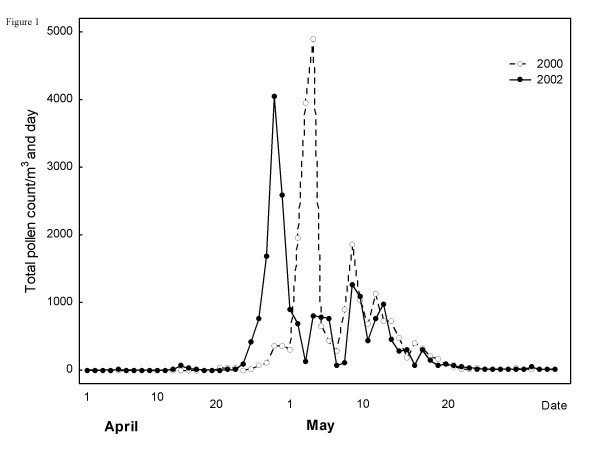
Pollen counts, grains/m^3^, during the birch pollen season in 2000 and 2002.

The number of airborne pollen particles was counted by the Palynological Laboratory, Swedish Museum of Natural History, Stockholm, between 1 April and 31 May 2000 and 2002. Pollen recordings were made using a Burkhard seven-day recording volumetric spore trap [20]. The trap was placed on the roof of the Arrhenius Laboratory at Stockholm University, 20 m above the ground, in the centre of Stockholm. The pollen count is expressed as the mean number of pollen grains per day and per cubic metre of filtered air, at two-hour intervals during the day. The pollen counts during the two seasons were comparable, in terms of both the pollen peak and the duration of the season (Figure 1).

#### Skin prick tests

were performed with nine standard aeroallergen extracts (birch, timothy, mugwort, cat dander, dog dander, horse dander, *Dermatophagoides pteronyssinus, Cladosporium herbarum *and *Alternaria*), using Soluprick SQ ALK (Hörsholm, Denmark). The results were read after 15 minutes, measuring the largest diameter of the weal and its perpendicular diameter, and the product was expressed in mm^2^. Skin reactions were considered positive when larger than 9 mm^2^.

#### Spirometry

Lung function tests were performed with a Vitalograph-Compact spirometer, (Vitalograph Ltd., Buckingham, England). FEV_1_, FVC, FEV_1_/FVC% and PEFR were recorded. The reference values were those from European Community for Coal and Steel [21]. Peak flow rate (PEFR) values were measured by the subjects in the morning and in the evening during the pollen season using a mini-*Wright *Peak Flow Meter (Clement Clarke International Ltd., Essex, England) and recorded in the diary.

#### Nasal lavage

Lavage of the nasal mucosa was performed according to Wåhlinder *et al*. [22] with a 20-ml syringe attached to a nose olive. The subjects stood, with their heads flexed about 30° forward. At room temperature (20–22°), sterile 0.9% saline was introduced into the nasal cavity. Each nostril was lavaged with 5 ml of the solution, which was flushed back and forth five times via the syringe, at intervals of a few seconds. The recovered fluid was weighed and the amounts obtained were comparable in all subjects. The fluid was transferred into 10-ml polypropylene centrifuge tubes. It was kept on ice and, within 300 minutes, the solution was centrifuged at 800 *g *for five minutes. The supernatant was then centrifuged at 1,400 g for five minutes and immediately frozen in small aliquots to -70°C for analyses of eosinophilic cationic protein (ECP) and human neutrophil lipocalin (HNL). The cell suspension was concentrated in object glasses using cytospin centrifugation for differential counting.

#### Induced sputum

Sputum samples were obtained by hypertonic saline inhalation modified after Pizzichini *et al*. [23,24], except that the subjects were not pretreated with inhaled bronchodilators. FEV_1 _was measured before and after the inhalation of physiological saline and induction was then started with hypertonic saline solution. An ultrasonic nebuliser (OMRON U 1, Sonesta Tamro no 28 36 06, Stockholm, Sweden) was used for the inhalations. FEV1 was measured before and 90 seconds after inhalation of physiologic saline. Then hypertonic (4.5%) saline solution was administered for 0.5, 1, 4, 8 and 16 minutes. FEV1 was recorded 90 seconds after each inhalation. The subjects were asked to rinse their mouths with water and then to cough sputum into a sterile container. The test was stopped if FEV1 fell with 20% or more compared with the value obtained after inhaling isotonic saline. After completing the inhalation, the subjects were instructed to "huff" and cough into the container. The mucus clods were aspirated with a 2-ml syringe and collected. The entire sputum clod was weighed and immediately transported to the laboratory. The sputum sample was kept on ice and an equal amount of 0.2% dithiothreitol in phosphate buffer, sputolysin (CalbioChem, SputolysinReagent, art no 56000), was added before incubation at 22°C for 15 minutes. The sample was then centrifuged and the supernatant frozen at -70°C for the subsequent analysis of ECP and HNL. The cell suspension was concentrated on object glasses using cytospin centrifugation for differential cell counting.

### Cell count and specific inflammatory markers

Four ml of EDTA blood was collected for routine laboratory tests of eosinophil counts (Cell-Dyn 4000, Abbott). Serum for analyses of ECP and HNL was collected from the four ml of blood in SST tubes (Becton Dickinson AB), kept for 60 minutes in room temperature and centrifuged for 10 minutes at 3600 rpm. The serum was frozen to -70°C.

Blood eosinophil counts (B-Eos) (normal range 0.0–0.5 × 10^9^/l) were determined using routine methods at the Department of Clinical Chemistry, Uppsala University Hospital. Differential cell counts were obtained using a cytospin preparation (Cytospin, Shandon, Southern Instruments, Sewickley, USA) stained with May Grünewald and Giemsa, and examined under light microscope. ECP was analysed by Unicap, Pharmacia. HNL was assayed by a double-antibody RIA described in detail elsewhere [25]. The inter- and intra-assay coefficients of variations were < 10% for all tests. Specific IgE was determined with a RadioAllergoSorbent Test (RAST) at the Department of Clinical Immunology, Uppsala University Hospital (normal < 0.35 kU/L).

#### Diary

Starting two weeks prior to the season visit, the subjects with allergic rhinitis and allergic asthma recorded their morning PEFR and evening PEFR every day. The highest value of three was registered in the diary.

Symptoms were graded from 0 to 3 (none to severe) for each symptom: rhinitis, conjunctivitis and respiratory symptoms (shortness of breath, chest tightness, cough) during the day and night. A symptom score was constructed for rhinoconjunctivitis by adding the nose and eye score each day divided by two. The daytime and nocturnal respiratory score was combined in the same way. A total score for the four symptoms was also calculated. A mean symptom score for each subject was calculated from the sum of symptoms, divided by the number of registered days.

Medication was recorded in the diary. The following medication categories were used: oral anti-histamine, topical treatment in the nose and eyes (anti-histamines and/or cromones) and inhaled short-acting beta-agonists during the day and night. The number of drugs in each category was calculated for each day and divided by the number of registered days and a total medication score was generated by combining all the categories.

#### Ethics committee

The study was performed with the approval of the ethics committee at the Medical Faculty at Uppsala University and informed consent was obtained from each subject.

#### Statistical evaluation

The Kruskal-Wallis, ANOVA and Mann-Whitney U test were used to evaluate statistical differences between patient groups. For paired analyses, we used Friedman's ANOVA and Wilcoxon's matched pairs test. A p-value of < 0.05 was considered significant. All the calculations were performed using the Statistica statistical software package (Statsoft Inc, Tulsa, Oklahoma, USA).

## Results

No significant differences concerning sex, age or smoking were found between the three groups of test persons. There were no differences in allergy variables between the two groups of allergic patients. (Table 2).

**Table 2:** Characteristics at baseline (mean, range). No significant differences were found between any of the groups in terms of gender, age, smoking and lung function or between the two allergic groups in terms of allergy variables.

	Control group (n = 5)	Allergic rhinitis (n = 9)	Allergic asthma (n = 7)
Gender (male/female)	2/3	8/1	5/2
Age	38 (27–58)	43 (24–66)	44 (27–56)
Ex-smokers	0	2	1
SPT birch mm^2^	0	47.1 (20–88)	43.6 (26–64)
Specific IgE for birch kU/L	0	3.1 (2–4)	3.4 (2–5)
*Number of positive SPT	0	4 (0–4)	5 (1–6)
FEV_1 _(L)	3.59 (3.02–3.95)	4.0 (2.4–4.9)	3.41 (2.56–3.97)
FEV1 (% of predicted)	105 (88–125)	102 (75–139)	96 (83–108)
PEFR (L/min)	571 (348–854)	615 (415–826)	506 (347–652)
PEFR (% of predicted)	117 (84–169)	113 (82–140)	100 (73–133)

* In addition to birch

### Lung function, medication and symptoms

**Figure 2:**
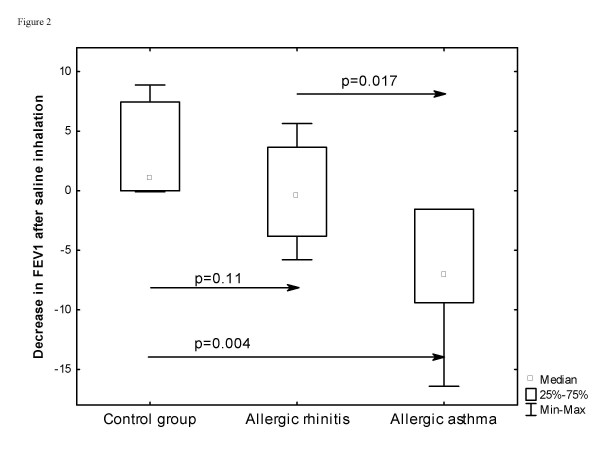
Difference in ΔFEV_1 _(change in FEV_1 _compared to baseline FEV_1_) between allergic rhinitis and allergic asthma after inhaling hypertonic saline solution (4.5%) at baseline.

At baseline no significant difference was found regarding lung function (Table 2), except that patients with allergic asthma had a significantly larger decline in FEV_1 _after inhaling hypertonic saline solution (median (IQ range): -7.0 (-9.4, -1.6)%) than patients with allergic rhinitis (-0.4 (-4.1, 4.0)%) or controls (1.1 (-0.5, 8.2)%) (Figure 2).

The group with allergic asthma had a significantly lower morning and evening PEFR during the birch pollen season (Table 3). No significant difference was found between the groups regarding symptoms or treatment for rhinoconjunctivitis. No significant changes in ΔFEV_1 _(change in % in FEV_1 _compared with before-season spirometry) could be seen during the pollen season in any of the allergic groups (Table 3).

**Table 3:** Diary recorded during birch pollen season (median, upper and lower quartiles).

	Allergic rhinitis	Allergic asthma	p-value
*Symptom score*			
Nose and eyes	2.4 (1.1, 3.9)	3.8 (1.9, 4.9)	0.37
Respiratory	1.3 (0, 4.2)	1.3 (0.4, 2.0)	0.83
Total	3.8 (2.4, 6.7)	4.2 (3.0, 6.9)	0.67

*Medication score*			
Oral anti-histamine	1 (1, 1)	1 (0, 1.1)	0.67
Topical *	1.75 (0.25, 2)	0.9 (0, 2.6)	0.83
Beta-2-agonists	0 (0, 0)	0.6 (0.2, 1.9)	0.006*
Total	3 (1.2, 3.5)	2.7 (0.5, 5.6)	0.79

*Lung function*			
Morning PEFR (L/min)	575 (550, 620)	475 (433, 551)	0.020
Evening PEFR (L/min)	610 (555, 630)	478 (449, 551)	0.005
ΔFEV_1 _**	-0.07 (-0.37, 0.2)	-0.21 (-0.43, -0.16))	0.26

* Topical medication: anti-histamines and/or cromones

** Change in FEV_1 _in % compared with before-season spirometry

### Inflammation

No significant differences in inflammatory markers in blood, nasal lavage or induced sputum were found between any of the groups at baseline, except for a significantly higher value for nasal lavage (NL) -ECP in the rhinitis group compared with the control group (p = 0.045) (Table 4).

**Table 4:** Baseline inflammatory parameters in blood, nasal lavage and induced sputum (median, upper and lower quartiles) (* = p < 0.05 compared with the control group)

	Control group	Allergic rhinitis	Allergic asthma
*Blood*			
B-eosinophils 10^9^/L	0.12 (0.10, 0.13)	0.1 (0.08, 0.17)	0.14 (0.11, 0.21)
S-ECP μg/L	8.6 (5.5, 9.4)	9.0 (6.2, 11.8)	6.1 (4.4, 11.3)
S-HNL μg/L	89 (56, 105)	93 (84, 99)	99 (65, 118)

*Nasal lavage*			
Eosinophils%	0 (0, 0)	0.1 (0–0.4)	0.3 (0, 0.5)
ECP μg/L	2 (2, 2)	3 (2, 13)*	2 (2, 5)
HNL μg/L	30 (26, 33)	74 (65, 176)	83 (40, 202)

*Sputum*			
ECP μg/L	149 (81, 280)	192 (126, 403)	146 (54, 350)
HNL μg/L	1724 (636, 2570)	839 (573, 1854)	1606 (480, 2357)

**Figure 3:**
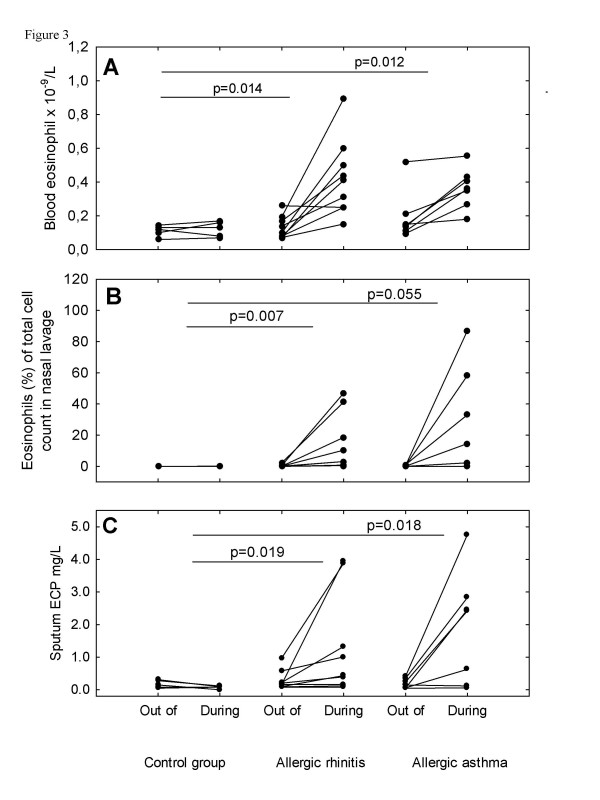
Blood eosinophil counts (A), Percentage of eosinophils in nasal lavage (B) and sputum ECP (C) increase during pollen season in both allergic rhinitis and patients with allergic asthma.

During the birch pollen seasons there were significant increases in B-Eos and sputum (Sp) ECP in the rhinitis and asthma groups but not in the control group (Table 5, Figure 3). The ΔNL-Eos was significantly larger in the rhinitis than in the control group (Table 5, Figure 3). Significantly larger decreases in both ΔNL-HNL and ΔSp-HNL were found in the group of subjects with predominant rhinitis than in the control group (Table 5). There were no differences between the two groups of allergic patients regarding the changes in inflammatory parameters during the pollen seasons.

**Table 5:** Changes in inflammatory parameters in blood, nasal lavage and induced sputum between baseline out of season and during birch pollen season (median, upper and lower quartiles).

	Control group	Allergic rhinitis	p-value	Allergic asthma	p-value
*Blood*					
Δeosinophils 10^9^/L	0.01 (0, 0.025)	0.27 (0.17, 0.43)	0.014*	0.17 (0.03, 0.27)	0.012*
ΔS-ECP μg/L	2,3 (0.5, 2.4)	9.6 (4.9, 16.1)	0.071	8.6 (-0.2, 11.5)	0.12
ΔS-HNL μg/L	19.1 (4.7, 31.4)	1.8 (-3.5, 5.3)	0.20	1,7 (-1.0, 14.4)	0.12

*Nasal lavage*					
Δeosinophils %	0.05 (0, 0.1)	6.7 (1.9, 28.8)	0.007*	23.3 (2.3, 57.8)	0.055
ΔECP μg/L	0 (0, 5)	1.4 (-0.1, 5.8)	1.0	4 (0, 10.1)	0.29
ΔHNL μg/L	85 (45, 109)	-17 (-44, 21.9)	0.039*	-10 (-28.8, 24.8)	0.22

*Sputum*					
ΔECP μg/L	-146 (-163, 0.3)	310 (19, 2000)	0.019*	2180 (12, 2480)	0.018*
ΔHNL μg/L	-766 (-1655, 302)	46 (-340, 190)	0.040*	-218 (-1084, 748)	0.17

## Discussion

The main finding in our study is that birch pollen allergic patients with either rhinitis or asthma as predominant expression of their disease have comparable levels of eosinophil inflammation in the blood, nose and lower airways both before and during the pollen season. Our results highlight the close relationship between the upper and lower airways in both allergic diseases and support the concept of "one airway – one disease" [26,27]. The results we obtained during seasonal allergen exposure are in accordance with the results of studies using allergen challenges, where Braunstahl *et al*. showed that both nasal provocation and segmental bronchial allergen challenge induce inflammation in both bronchial and nasal mucosa, i.e. the link is bi-directional [9,10]. Nasal allergen challenge tests have also produced data supporting the concept of "allergy syndrome", showing eosinophil inflammation in the oesophagus [28], and other studies have showed eosinophil inflammation in the stomach and small intestine during the pollen season, despite a lack of gastrointestinal symptoms [29].

In spite of the fact that both groups of allergic patients had a similar inflammation pattern, distinct differences were found between the groups. The patients with predominant asthma had a higher bronchial responsiveness when measured with hypertonic saline challenge, a lower PEFR during the season and were much more likely to use β_2_-agonists than patients with predominant rhinitis. On the other hand, both groups had the same degree of rhinoconjunctivitis and used the same amounts of oral antihistamines and local treatment in eyes and nose. Our data partly disagrees with that of Ciprandi *et al*. [30], who found a decrease in FEV1 during the pollen season in rhinitis patients, whereas, in Ciprandi's study, patients showing early bronchial impairment and BHR patients with perennial rhinitis and diagnosed BHR were included.

The eosinophil inflammation in different compartments, measured as the number of eosinophils as well as the concentration of ECP, was comparable between the two allergic groups. In both groups, a significant increase was found in B-Eos and Sp-ECP compared with the non-allergic controls, whereas no distinct pattern was found when it came to neutrophil inflammation. The role of neutrophils in mild asthma and allergic rhinitis is uncertain, whereas in severe asthma neutrophils play an important role in the pathogenesis of airway inflammation [31]. Our data confirm that there is an eosinophil inflammation in both the nasal and bronchial mucosa and systemically in both allergic asthma and allergic rhinitis during the pollen season. However, in spite of the same level of eosinophil inflammation in the lower airways, only the asthmatic group present with significant BHR and impairment of lung function during the pollen season. These data are consistent with reports from other groups [32,33], indicating that other factors, such as airway remodelling, play a role in the development of BHR in asthma. Boulay *et al*. also demonstrated an increase in eosinophils in induced sputum after repeated very-low-dose allergen challenge in allergic rhinitis, without any changes in FEV1 or response to metacholine challenge [19].

The strength of our study is that we have evaluated the nasal and bronchial inflammation in the same patients simultaneously. Another advantage is that we have used natural exposure to allergens. Allergen exposure during the pollen season is a low-dose challenge over a long period and is more like the natural course of allergy development than high-dose allergen challenge. It is known from both experimental studies and real life that very high doses of allergen can elicit asthma symptoms in non-asthmatics, if the doses are high enough [34]. The Swedish birch pollen season is very convenient to study, as it is relatively short and defined in time and comes after a long, cold winter, making it a suitable model for seasonal allergy studies. Usuallay the birch pollen season varies in pollen counts, with high pollen counts every second year. One drawback of this study is the small number of subjects in each group, which limited the opportunity to find differences between the two allergic groups.

In conclusion allergic asthma and allergic rhinitis have the same degree of eosinophil inflammation in blood, nasal mucosa and bronchial mucosa during the pollen season. In spite of this, only the asthmatic group present with significant BHR and impairment of lung function during the pollen season. This indicates that eosinophil inflammation in the bronchial mucosa alone is not enough to cause asthma and that factors other than eosinophil inflammation determine whether or not an allergic patient develops asthma.

## Abbreviations

• B-Eos: blood eosinophil count

• ECP: eosinophil cationic protein

• FEV1: forced expiratory volume in 1 second

• FVC: forced vital capacity

• HNL: human neutrophil lipocalin

• NL: nasal lavage

• PEFR: peak expiratory flow rate

• RAST: radio-allergo-sorbent test

• Sp: sputum
